# Centrally mediated pain features predict persistent pain in early inflammatory arthritis: a 2-year prospective study

**DOI:** 10.1097/j.pain.0000000000004099

**Published:** 2026-09-02

**Authors:** Zoe Rutter-Locher, Sam Norton, Bina Menon, Tom Esterine, Ruth Williams, Leonie S. Taams, Bruce W. Kirkham, Kirsty Bannister

**Affiliations:** a Rheumatology Department, Guy's and St Thomas' NHS Trust, London, United Kingdom; b Department of Inflammation Biology, School of Immunology & Microbial Sciences, Faculty of Life Sciences and Medicine, King's College University, London, United Kingdom; c Centre for Rheumatic Diseases, King's College London, London, United Kingdom; d Department of Life Sciences, South Kensington Campus, Imperial College London, London, United Kingdom

**Keywords:** Arthritis, Pain trajectory, Longitudinal, Patient-reported outcome measures

## Abstract

Patients with inflammatory arthritis (IA) often report pain not necessarily coupled to active inflammation. We previously demonstrated that a significant subset of patients with newly diagnosed IA report features of centrally mediated pain. In this prospective study following the same IA cohort, we tracked the relative contributions of inflammatory/noninflammatory mechanisms to pain over 2 years. We used patient-reported outcome measures (PROMs), clinical data (including ultrasound), and standardised quantitative sensory testing. Mixed-effects regression models examined longitudinal associations. We recruited 66 patients. At 6, 12, and 24 months, 62%, 57%, and 49% of individuals, respectively, reported persistent pain (numerical rating scale ≥3). Using factor-derived composite scores, baseline and longitudinal inflammation was not associated with subsequent pain (*b* −0.05, *P* = 0.87), whereas higher centrally mediated pain markers predicted less pain reduction (*b* 1.41, 95%, *P* < 0.001). Baseline PROMs of centrally mediated pain (fibromyalgia severity average marginal effect [AME]: 0.94, *P* < 0.001, painDETECT AME: 1.03, *P* < 0.001) and mental health (eg, Generalised Anxiety Disorder-7 AME: 1.08, *P* < 0.001) were the strongest predictors of persistent pain. Baseline trapezius pressure pain threshold (PPT) and conditioned pain modulation (CPM) pain detection threshold (PDT) effect showed modest association (trapezius PPT AME: −0.51, *P* = 0.054, CPM PDT effect AME: −0.49, *P* = 0.051) whereas temporal summation of pain (AME: 0.09, *P* = 0.756) and CPM pain tolerance threshold effect (AME: −0.01, *P* = 0.964) did not. In our cohort, persistent pain was associated with higher baseline and longitudinal scores for centrally mediated pain according to PROMs rather than inflammation according to joint ultrasound. In IA, early identification and treatment of features of centrally mediated pain may improve pain-related outcomes.

## Abstract 2

Supplemental Digital Content is Available in the Text.

Pain persistence in arthritis is not necessarily explained by inflammation but rather baseline and longitudinal scores for patient-reported outcome measures of centrally mediated pain.

## 1. Introduction

Approximately 30% of patients with inflammatory arthritis (IA) have persistent, unacceptable pain,^6,17,37,43^ consistently reported as a major concern above other symptoms, including fatigue, swelling, and stiffness.^7,23,32,39^ This pain is strongly associated with physical disability and reduced ability to participate in social activities^29^ and the workforce,^24^ and is closely linked to psychological distress (including depression and anxiety) and poorer treatment adherence and outcomes.^1,46^ A failure to understand the mechanisms that underpin pain in IA has led to ineffective analgesic approaches, including increased opioid prescribing and inappropriate escalation of immunosuppressive therapy, adding burden to healthcare systems.^45^

In established IA, persistent pain arises from a complex interplay between ongoing inflammatory and noninflammatory maladaptive processes.^19,36^ Growing evidence indicates that pain can persist even in the absence of inflammation and/or that pain levels may not correlate with the level of inflammation.^14,17^ We recently conducted a systematic review evidencing that up to 42% of patients with IA report pain sensitisation and neuropathic-like pain,^31^ suggesting the involvement of dysregulated peripheral and/or central nervous system sensory processing beyond synovial inflammation. Consistent findings of greater widespread pain sensitivity, as measured by nonarticular pressure pain thresholds in patients with IA compared with healthy individuals,^41^ further support this involvement.

Although there is growing recognition of pain as a major problem in IA, research focused on pain and its trajectory from disease diagnosis is limited. This gap in knowledge is a problem because the early stage of disease could offer a critical window of opportunity for analgesic intervention. We recently recruited a cohort of patients with newly diagnosed IA, all reporting pain scores of ≥3 on the numerical rating scale. We assessed their pain through clinical evaluation, patient-reported outcomes, musculoskeletal ultrasound, and quantitative sensory testing incorporating pressure pain thresholds and conditioned pain modulation (CPM). Despite active peripheral joint inflammation, a significant proportion additionally exhibited features suggestive of centrally mediated pain processing (ie, fibromyalgia criteria, neuropathic-like pain according to painDETECT questionnaire, and a mean CPM effect for pain detection thresholds below that found in healthy populations).^34^

In this study, using these previously reported baseline findings as our results foundation, we present a longitudinal analysis of features associated with different pain phenotypes in a real-world IA cohort. Precisely, we followed the same patients over a 24-month period with (additional to baseline) assessments at 6, 12, and 24 months to prospectively investigate the evolving contributions of inflammatory vs noninflammatory (ie, features suggestive of peripherally vs centrally mediated) pain. Our aim was to identify, as inferred by clinical, psychological, and sensory assessments incorporating disease activity, quality of life, mental health status, and pain characteristics, the trajectory of features associated with persistent pain in our IA patient population.

## 2. Methods

### 2.1. Study design and ethical approval

The “Pain phenotypes and their Underlying Mechanisms in Inflammatory Arthritis” (PUMIA) study is a single-site, prospective observational study designed to investigate pain mechanisms in patients with IA. Patients with newly diagnosed IA were recruited (as soon after diagnosis as possible) and followed up at 6, 12, and 24 months. All participants provided written informed consent, and the study received ethical approval from the Bromley Research Ethics Committee and the Health Research Authority (REC reference: 21/LO/0712).

### 2.2. Participant recruitment and assessments

Individuals who reported a total numerical rating scale (NRS) pain score of 3 or higher on a scale of 0 to 10, received a clinician diagnosis of peripheral inflammatory joint disease, and experienced symptom onset within the past 12 months were recruited. Patients were enrolled in the study as soon as feasible after their diagnosis. Full recruitment criteria are available in the supplementary material (Supplementary Data S1, https://links.lww.com/PAIN/C559). At the baseline study visit^35^ and 12 months, participants underwent in-person assessments, which included assessment of (1) pain and quality of life: pain ratings on a 0 to 10 NRS, of both overall pain and pain in their most affected joint, in the previous 24 hours, and the Musculoskeletal Health Questionnaire (MSK-HQ) for quality of life; (2) clinical inflammatory disease activity: high-resolution musculoskeletal ultrasound (HRUS: 14 joints assessed for power Doppler activity [PD]), CRP, 28-TJC, 28-SJC, and DAS28-CRP; (3) psychological distress and features suggestive of centrally mediated pain: patient-reported outcome measures (PROMs) including the Patient Health Questionnaire-9 (PHQ-9) for depression, the Generalised Anxiety Disorder-7 (GAD-7) for anxiety, the Patient Health Questionnaire-15 (PHQ-15) for somatic symptom burden, the 2016 revision of the American College of Rheumatology (ACR) 2010 modified diagnostic criteria for fibromyalgia (comprising the Widespread Pain Index [WPI] and the Symptom Severity Scale [SSS], fibromyalgia severity was calculated as the sum of the WPI and SSS scores), and the painDETECT questionnaire (a validated screening tool for neuropathic-like pain, commonly used as a proxy for identifying centrally mediated pain due to the similarity in symptom profiles), and (4) pressure pain thresholds and dynamic quantitative sensory testing (QST) incorporating temporal summation of pain (TSP), and conditioned pain modulation (CPM), all according to previously reported protocols^30,35^ (protocols detailed in supplementary data S1, https://links.lww.com/PAIN/C559). At 6 and 24 months, participants completed a remote follow-up consisting of pain ratings and PROMs (MSK-HQ, PHQ-9, GAD-7, PHQ-15, fibromyalgia criteria and severity, and painDETECT).

### 2.3. Sample size calculation

Our sample size was based on the hypothesis that peripheral inflammatory pain would predominate at diagnosis, but that, over disease course, noninflammatory (supposed centrally mediated) pain mechanisms would contribute—for some individuals—to the pain experience. A target sample size of 60 was calculated based on providing acceptable balance between feasibility of recruitment and precision in the estimate of the proportion of people transitioning from an inflammatory-driven pain phenotype to an inferred noninflammatory centrally mediated pain phenotype (expected to be 30% based on literature),^31^ where the maximum width of the 95% CI was ±12.8%. Although relatively wide, we considered this a feasible number to recruit and acceptable when considered alongside the use of continuous scores to provide additional contextual information in addition to the dichotomised variable.

### 2.4. Statistical analysis

All data are presented as mean ± standard deviation (SD). Absolute reference data (matched for age, sex, and site, being the dorsum of the hand for MDT, MPT, and WUR, and thenar eminence for pressure pain threshold [PPT]) were used to normalise test results of the individual participant by calculating the z transformation = (value participant − mean controls)/SD controls.^29^ A z-score beyong ±1.96 is considered extreme, falling outside the range of 95% of the healthy population.

#### 2.4.1. Pain trajectories

Longitudinal changes in continuous outcomes, including pain, inflammatory measures, indicators of centrally mediated pain, and mental health and quality of life, were analysed using linear mixed-effects regression models, with a random intercept to account for repeated measures within participants over time. Time was included as a dummy-coded categorical fixed effect allowing for the estimation of time point–specific marginal means with 95% confidence intervals. The approach used allows for the inclusion of participants with at least one assessment of the outcome under a missing at random assumption, conditional on variables associated with missingness being included in the model. Pairwise comparisons between time points were performed with Bonferroni correction. For binary outcomes (eg, proportion reporting pain ≥3), mixed-effects logistic regression was used, with predicted probabilities over time obtained from the model. To enable direct comparison of change over time across variables measured on different scales, all continuous variables were standardised to baseline values. For each variable, the mean and SD at baseline were calculated, and z-scores were computed at each subsequent time point as (x-baseline mean)/baseline SD.

Given that we had numerous measures, we used exploratory factor analysis (principal factoring) to generate 2 composite scores: one composite score was extracted from inflammation-related variables (DAS28-swollen joint count, C-reactive protein, evaluator global assessment, and HRUS-PD score) and another from centrally mediated pain-related variables (painDETECT, fibromyalgia severity, PHQ-9, and pressure pain threshold at right trapezius). An a priori decision was made to exclude TSP and CPM from this composite as these measures infer specific neurophysiological processes, whereas we wished to capture the broader clinical phenotype captured by the remaining variables.

The relationship between inflammation-related and centrally mediated pain–related composite scores and residual change in pain was examined using separate linear mixed-effects models, with random intercepts for participants and with pain as the outcome variable. These models controlled for composite inflammation or centrally mediated pain composite scores at the previous time point, prior pain, age, sex, and time.

#### 2.4.2. Predictors of persistent pain

Baseline characteristics were compared between participants with and without clinically relevant pain (NRS ≥ 3) at 6, 12, and 24 months using 2-sided independent *t* tests. For each variable, we report the mean difference (95% CI) and effect sizes using Cohen's *d* (95% CI). Effect sizes were interpreted using standard thresholds: 0.2 (small), 0.5 (medium), and 0.8 (large). Exploratory mixed-effects linear regression models were then used to examine whether baseline predictors (factor-derived central and inflammatory composite scores, as well as individual baseline variables) were associated with subsequent pain, accounting for repeated measures and including time-by-predictor interaction terms. The variance in pain explained by each predictor was quantified using Nakagawa's marginal R^2^, calculated as the increase in marginal R^2^ when the predictor was added to a baseline model containing age, sex, time, and a random participant intercept. For all analyses, a *P*-value <0.05 was considered statistically significant. Analyses were conducted in Stata V17.0 (StataCorp, College Station, TX) and R Studio using R version 4.5.0 (2025-04-11).

## 3. Results

### 3.1. Study cohort and follow-up completion

Building on our previously published baseline cohort of 61 patients^35^ and due to ongoing recruitment at the time of baseline data publication, the present study sample size was 66. Mean age was 50 years, 56% were female, and ethnicity were diverse (Table 1). The majority were diagnosed with rheumatoid arthritis (Table 1). Mean time since diagnosis was 1.2 months. At baseline, patients had active disease (mean DAS28-CRP 3.9) and active synovitis on examination ±MSK US (65 with ≥1 active joint). The mean overall pain score was 5.4/10, and 67% and 62% of patients screened positive for depression and anxiety, respectively. 20 to 30% of patients fulfilled criteria for widespread pain, fibromyalgia, and likely neuropathic-like pain on painDETECT questionnaire. For QST, on average, PPT were lower at joint sites than at trapezius (*P* < 0.05). The mean TSP was 1.9 and the mean CPM effect was lower than that in healthy individuals (CPM effect for pain detection threshold [PDT] 2.6 and pain tolerance threshold [PTT] 3.4) (Table 1, Supplementary Table S1, https://links.lww.com/PAIN/C559). Follow-up rates were acceptable across the study period, with 61 (92%) of participants completing at least one follow-up assessment. Data were available for 53 participants (80%) at 6 months, 58 (88%) at 12 months, and 57 (86%) at 24 months. Participants lost to follow-up had higher baseline DAS28 tender joint counts and PHQ-9 scores than those who completed 24-month follow up (Supplementary Table S7, https://links.lww.com/PAIN/C559).

**Table 1:** Selected baseline characteristics of the cohort (n = 66).

Characteristic	Value (n = 66)
Demographics	
Age, y, mean (SD; range)	50 (15; 19-86)
Female, n (%)	37 (56)
Ethnicity, n (%)	
White British	36 (55)
Black or Black British	11 (17)
Asian or Asian British	9 (14)
Other	10 (15)
Disease characteristics	
Diagnosis, n (%)	
Rheumatoid arthritis	54 (82)
Psoriatic arthritis	10 (15)
Spondyloarthritis	2 (3)
Time since diagnosis, mo, mean (SD)	1.2 (2.2)
Musculoskeletal ultrasound	
≥1 GS-positive, ≥1 PD-positive, or ≥1 SJC, n (%)	65 (99)
Mental health	
GAD-7, mean (SD)	7.4 (6.0)
Any anxiety (≥5), n (%)	41 (62)
PHQ-9, mean (SD)	8.3 (6.3)
Any depression (≥5), n (%)	44 (67)
PHQ-15, mean (SD)	8.9 (5.3)
Any somatic symptoms (≥5), n (%)	53 (80)
Pain	
Overall body pain (NRS 0-10), mean (SD)	5.4 (2.1)
Affected joint pain (NRS 0-10), mean (SD)	5.1 (2.5)
Widespread pain and fibromyalgia features	
Widespread pain index (WPI), mean (SD)	5.6 (2.8)
WPI ≥ 7, n (%)	21 (32)
Fibromyalgia (2016 ACR criteria), n (%)	15 (23)
Fibromyalgia severity (WPI + SSS), mean (SD)	10.9 (4.9)
Tender–swollen joint count difference ≥7, n (%)	15 (23)
Neuropathic-like pain	
painDETECT, mean (SD)	12.7 (7.7)
Likely neuropathic-like (≥19), n (%)	15 (23)
Quantitative sensory testing, mean (SD)	
TSP ratio	1.9 (1.2)
CPM PDT effect (kPa)	2.6 (8.3)
CPM PTT effect (kPa)	3.4 (6.7)

Full demographics, disease characteristics, and additional measures are reported in Supplementary Table S1, https://links.lww.com/PAIN/C559.

ACR, American College of Rheumatology; CPM, conditioned pain modulation; GAD-7, Generalised Anxiety Disorder Scale; GS, grayscale; NRS, numerical rating scale; PD, power Doppler; PDT, pain detection threshold; PHQ-15, Patient Health Questionnaire–Somatic Symptoms; PHQ-9, Patient Health Questionnaire–Depression; PTT, pain tolerance threshold; SJC, swollen joint count; SSS, symptom severity scale; TSP, temporal summation of pain; WPI, widespread pain index.

### 3.2. Pain persists in 49% of participants at 24 months

**Figure 1.**
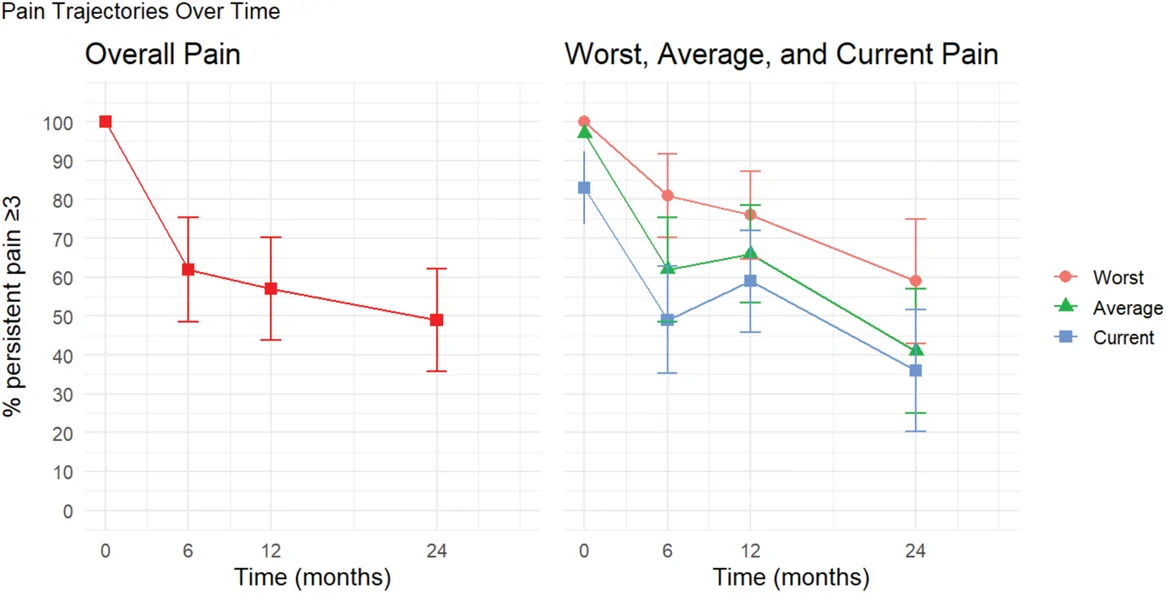
Pain trajectories over time in early inflammatory arthritis. The figure shows the percentage of participants reporting persistent pain (score ≥3 on 0-10 numerical rating scale) over 24 months. Left panel shows overall pain; right panel shows worst, average, and current pain scores at each time point, from painDETECT questionnaire. Although overall pain improved, 49% of patients continued to report clinically relevant pain. Total N at each time point: baseline (n = 66), 6 months (n = 53), 12 months (n = 58), and 24 months (n = 57). Error bars represent 95% confidence intervals.

At baseline, all participants reported persistent pain ≥3.^35^ The proportion who reported persistent pain ≥3 at 6, 12, and 24 months was 62% (95% CI: 48-74%), 57% (95% CI: 44-69%), and 49% (95% CI: 36-61%), respectively (Fig. 1). Of note, pain did not improve, or worsened, in 25% of participants over the two-year follow-up period. Pain score changes showed considerable individual variation (mean −2.2, SD 3.1; range −9 to +7). Specifically, the mean pain score reduced from 5.4 (SD 2.1) at baseline, to 4.1 (2.8) at 6 months, 3.7 (2.8) at 12 months, and 3.1 (3.1) at 24 months. Using a mixed-effects linear regression model, there was a significant reduction in pain between baseline and 6 months (*P* < 0.01). The further gradual decreases between subsequent consecutive time points did not reach statistical significance (Fig. 1). According to the painDETECT questionnaire, at 24 months, 36% of patients (95% CI: 25-49%) reported current pain ≥3, 41% (95% CI: 29-54%) average pain ≥3, and 59% (95% CI: 46-71%) worst pain ≥3 over the previous 4 weeks (Fig. 1). Using a mixed-effects logistic regression model, the likelihood of overall pain ≥3 declined over time: 64% at 6 months (95% CI: 52-77%), 57% at 12 months (95% CI: 44-70%), and 50% at 24 months (95% CI: 37-63%). These gradual decreases did not reach statistical significance.

### 3.3. Trajectories of inflammatory and centrally mediated pain markers over 24 months

#### 3.3.1. Markers of inflammation and patient-reported outcome measures of centrally mediated pain and mental health improved

**Figure 2.**
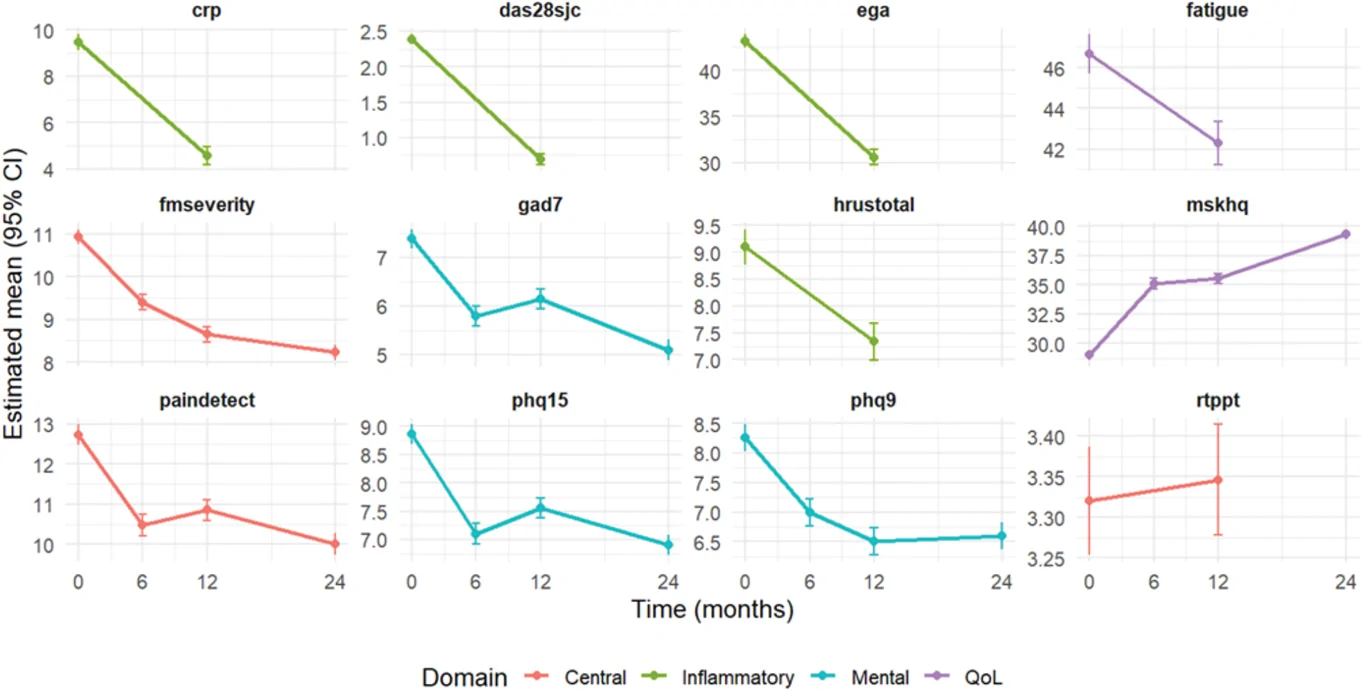
Modelled trajectories of centrally mediated pain, mental health and inflammatory measures over 24 months. Graphs show estimated marginal means (±95% confidence intervals) for 12 measures across 4 domains: centrally mediated pain (orange), mental health (blue), inflammation (green), and quality of life (purple). Results are derived from mixed-effects linear regression models with time as a fixed effect and participant ID as a random intercept. Each panel shows predicted outcome values at 0, 6, 12, and 24 months. Markers of centrally mediated pain reduced over time: painDETECT scores significantly decreased from 12.7 at baseline to 10.0 at 24 months, with modelled reductions at all follow-up points compared with baseline (*P* = 0.014 at 24 months). Fibromyalgia Severity Scores fell from 10.9 at baseline to 8.2 at 24 months, with significant reductions at all time points (*P* = 0.031 to *P* < 0.001). Trapezius PPT z-scores remained stable over time, with no significant change between baseline and 12 months (*P* = 0.904). Mental health scores reduced over time: PHQ-9 reduced from 8.3 to 6.6, with significant reductions at 12 and 24 months (*P* = 0.016 and *P* = 0.034, respectively). GAD-7 decreased from 7.4 at baseline to 5.1 at 24 months, with significant reductions at 6 months (*P* = 0.021) and 24 months (*P* < 0.001). PHQ-15 reduced from 8.9 to 6.9, with all time points significantly lower than baseline (all *P* < 0.05). Inflammatory markers reduced: CRP levels reduced from 9.5 mg/L at baseline to 4.6 mg/L at 12 months (*P* = 0.001). DAS28-SJC decreased from 2.4 to 0.7 (*P* < 0.001), and EGA from 43.2 to 30.6 (*P* < 0.001). MSK ultrasound (HRUS PD) scores reduced from 9.1 to 7.3, although this was not statistically significant (*P* = 0.096). Quality-of-life (QoL) measures improved significantly, with MSK-HQ scores increasing from 29.0 at baseline to 39.3 at 24 months (*P* < 0.001). However, fatigue scores showed a small, nonsignificant reduction from 46.7 to 42.3 (*P* = 0.30). CRP, C-reactive protein; DAS28SJC, disease activity score swollen joint count (28 joints); EGA, evaluator global assessment; FM, fibromyalgia; GAD-7, Generalised Anxiety Disorder Scale; HRUS, high-resolution ultrasound; MSK-HQ, Musculoskeletal Health Questionnaire; PHQ-9, Patient Health Questionnaire–Depression; PHQ-15, Patient Health Questionnaire–Somatic Symptoms; RTPPT, pressure pain threshold at right trapezius.

Modelled estimates of longitudinal change showed improvement in inflammatory features (CRP −4.90; DAS28-SJC −1.70; EGA −12.60) and patient reported outcome measures (PROMs) informed centrally mediated pain features (fibromyalgia severity −2.71; painDETECT −2.71) and mental health (GAD-7 −2.31; PHQ-9 −1.66; PHQ-15 −1.93) (Fig. 2, Supplementary Table S2, Supplementary Table S3, https://links.lww.com/PAIN/C559). Musculoskeletal Health Questionnaire scores improved significantly (+10.29), more than the minimal clinically important difference.^28^ Fatigue showed a nonsignificant reduction (−4.37).

#### 3.3.2. Joint sensitivity improved but quantitative sensory testing markers of centrally mediated pain did not change

Modelled estimates of longitudinal change in joint PPT increased (target joint PPT 0.72, right wrist 0.87, left wrist 0.64), indicating a reduction in peripheral joint sensitivity. There was no significant change in trapezius PPT (right trapezius 0.02, left trapezius −0.002) or in mean TSP or CPM effects (TSP 0.95, CPM PDT effect 2.13, CPM PTT effect −0.86) (Supplementary Table S3, https://links.lww.com/PAIN/C559).

### 3.4. Features of centrally mediated pain, but not inflammation, were associated with less pain improvement over time

**Figure 3.**
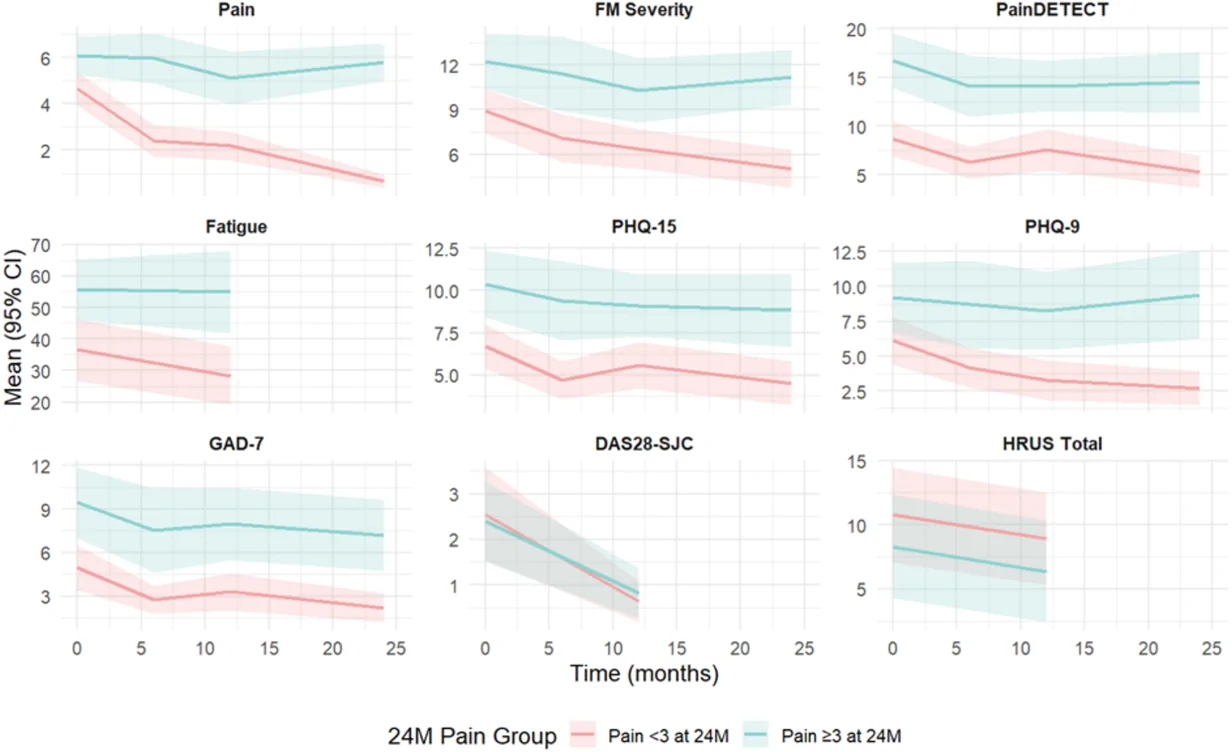
Trajectories of clinical measures, stratified by 24-month pain outcome. Graphs show mean scores (±95% CI) over time for participants with low pain (NRS < 3) vs persistent pain (NRS ≥ 3) at 24 months. Those in pain at 24 months had consistently reported pain over disease course, but those without pain had reduced their pain from baseline. The persistent pain group had consistently higher levels of FM severity and psychological distress, but inflammation (DAS28-SJC) reduced comparably in both groups. DAS28SJC, disease activity score swollen joint count (28 joints); FM, fibromyalgia; NRS, numerical rating scale.

52% (95% CI 33-70) of individuals with persistent pain (≥3/10) had no inflammation (no PD signal in any joint) at 12 months. As shown in Figure 3, despite similar baseline inflammation and comparable reductions in inflammation over time, individuals with persistent pain at 24 months had consistently higher levels of PROMs of centrally mediated pain across all time points (Fig. 3). Disease-modifying treatment was comparable between participants with persistent vs resolved pain at 24 months (csDMARD 93% vs 97%; bDMARD/tsDMARD 25% vs 21%), whereas analgesic and amitriptyline use was consistently higher throughout follow-up in those with persistent pain (Supplementary Tables S5 and S6, https://links.lww.com/PAIN/C559). Using composite scores and modelling residualised change in pain (ie, controlling for prior pain) over 24 months, inflammation at the previous time point was not associated with subsequent pain (*b*= −0.05, 95% CI −0.67 to 0.56, *P* = 0.87). However, centrally mediated pain at the previous time point was strongly and consistently associated with higher subsequent pain relative to expected pain, given the prior level (*b* = 1.41, 95% CI 0.83-1.99, *P* < 0.001), consistent with centrally mediated pain being associated with less improvement in pain over time. These relationships were observed across all time points (inflammation: 6 months: *b* = 0.20, 95% CI −0.59 to 0.99, *P* = 0.62; 24 months: *b* = −0.34, 95% CI −1.11 to 0.44, *P* = 0.39, central: 6 months: *b* = 1.59, 95% CI 0.83-2.36, *P* < 0.001; 12 months: *b* = 1.23, 95% CI 0.44-2.02, *P* = 0.002; 24 months: *b* = 1.39, 95% CI 0.59-2.20, *P* = 0.001).

### 3.5. Baseline predictors of persistent pain

#### 3.5.1. Baseline patient-reported outcome measures of centrally mediated pain and mental health, but not inflammation, predicted persistent pain

Individuals with pain ≥3 at 6, 12, and 24 months had consistently higher baseline centrally mediated pain and mental health markers, including fatigue (6 months: d = −1.08, *P* = 0.001; 12 months: d = −0.85, *P* = 0.002; 24 months: d = −0.74, *P* = 0.007), painDETECT (6 months: d = −1.04, *P* = 0.001; 12 months: d = −0.96, *P* < 0.001; 24 months: d = −1.28, *P* < 0.001), and fibromyalgia severity (6 months: d = −1.02, *P* = 0.001; 24 months: d = −0.74, *P* = 0.007, although not significant at 12 months: d = −0.48, *P* = 0.073). They also had worse mental health, including GAD-7 (6 months: d = −0.84, *P* = 0.005; 12 months: d = −0.83, *P* = 0.003; 24 months: d = −0.83, *P* = 0.003), PHQ-15 (6 months: d = −1.11, *P* = 0.001; 12 months: d = −1.08, *P* < 0.001; 24 months: d = −0.82, *P* = 0.003), and PHQ-9 (6 months: d = −0.98, *P* = 0.001; 12 months: d = −0.67, *P* = 0.014; 24 months: d = −0.53, *P* = 0.05), and worse quality of life on the MSK-HQ (6 months: d = 0.84, *P* = 0.005; 24 months: d = 0.79, *P* = 0.004, although not significant at 12 months: d = 0.27, *P* = 0.32). There were no consistent baseline differences between those with and without pain ≥3 in demographic variables, symptom duration, or inflammatory measures (Supplementary Table S4, https://links.lww.com/PAIN/C559).

Using composite scores, baseline inflammation was not associated with subsequent pain (*b* = 0.05, 95% CI −0.50 to 0.59, *P* = 0.87), whereas baseline centrally mediated pain was strongly and consistently associated with higher subsequent pain (*b* = 1.32, 95% CI 0.83-1.82, *P* < 0.001). These relationships were observed across all time points (inflammation: 6 months: *b* = 0.24, 95% CI −0.56 to 1.04, *P* = 0.56; 12 months: *b* = −0.19, 95% CI −0.94 to 0.56, *P* = 0.62; 24 months: *b* = −0.37, 95% CI −1.12 to 0.38, *P* = 0.33. Centrally mediated pain: baseline: *b* = 0.92, 95% CI 0.25-1.59, *P* = 0.007; 6 months: *b* = 1.81, 95% CI 1.04-2.58, *P* < 0.001; 12 months: *b* = 1.19, 95% CI 0.47-1.92, *P* = 0.001; 24 months: *b* = 1.46, 95% CI 0.73-2.19, *P* < 0.001). The relative contribution of each composite to explained variance reinforced these findings: adding the inflammation composite to a covariate-only model (age, sex, and time) increased the marginal R^2^ by 1.2% (from 14.5% to 15.7%), whereas adding the centrally mediated pain composite increased the marginal R^2^ by 14.8% (from 14.5% to 29.3%). A model including both composites explained 30.5% of the variance in subsequent pain (marginal R^2^).

When examining individual variables, PROMs of centrally mediated pain (painDETECT and fibromyalgia severity), somatic symptom burden (PHQ-15), and psychological distress (PHQ-9 and GAD-7) were the strongest predictors of subsequent pain, each showing large and consistent associations. Each of these measures independently added 10.8 to 16.6% to the variance in pain explained over and above age, sex, and time. Tender-swollen joint count difference showed a smaller but significant effect (Table 2).

**Table 2:** Standardised baseline predictors of subsequent pain.

Predictor	AME (per 1 SD)	95% CI	*P*	Marginal R^2^ (added)*
Mental health and somatic symptoms				
PHQ-15	1.23	0.78 to 1.69	**<0.001**	**15.8%**
Fatigue (VAS)	1.15	0.74 to 1.56	**<0.001**	**16.6%**
GAD-7	1.08	0.64 to 1.53	**<0.001**	**14.0%**
PHQ-9	1.01	0.54 to 1.47	**<0.001**	**12.0%**
Features associated with centrally mediated pain				
painDETECT	1.03	0.61 to 1.45	**<0.001**	**13.1%**
Fibromyalgia severity	0.94	0.49 to 1.38	**<0.001**	**10.8%**
Tender–swollen joint count difference	0.58	0.08 to 1.08	**0.023**	**4.2%**
PPT, right trapezius	−0.51	−1.02 to 0.01	0.054	3.0%
CPM PDT effect	−0.49	−0.97 to 0.00	0.051	5.0%
CPM PTT effect	−0.01	−0.52 to 0.50	0.964	2.1%
TSP ratio	0.09	−0.47 to 0.65	0.756	0.4%

Results from mixed-effects linear regression models showing average marginal effects (AMEs) of each predictor on pain, expressed per 1 SD increase in each predictor. Models were adjusted for age and sex, with repeated measures accounted for by random intercepts. Positive AMEs indicate higher subsequent pain; negative AMEs indicate lower subsequent pain. Predictors are grouped by domain. Cells where *P* < 0.05 are shaded bold.

* Baseline marginal R^2^ (covariates only—age, sex, and time) = 14.5%.

CPM, conditioned pain modulation; FM, fibromyalgia; GAD-7, Generalised Anxiety Disorder Scale; PDT, pain detection threshold; PHQ-9, Patient Health Questionnaire 9–Depression; PHQ-15, Patient Health Questionnaire 15–Somatic Symptoms; PPT, pressure pain threshold; PTT, pain tolerance threshold; RTPPT, right trapezius pressure pain threshold; TJC-SJC diff, tender minus swollen joint count 28 joints; TSP, temporal summation of pain.

### 3.6. Associations between baseline quantitative sensory testing markers and persistent pain were modest and inconsistent

Individuals in pain ≥3 at 6 and 24 months did not have significantly different baseline joint PPT from those who did not have pain (6 months; d = 0.20, *P* = 0.48, 24 months; d = 0.54, *P* = 0.05). Joint PPT was lower in those with persistent pain at 12 months (d = 0.56, *P* = 0.04). Baseline right wrist PPT was lower in those with persistent pain at 6 months (d = 0.65, *P* = 0.036) but not at 12 months (d = 0.16, *P* = 0.581) or 24 months (d = 0.33, *P* = 0.245). Left wrist PPT was not significantly different at 6 months (d = 0.45, *P* = 0.141) or 12 months (d = 0.12, *P* = 0.682) but was lower in those with persistent pain at 24 months (d = 0.59, *P* = 0.043).

Individuals with pain ≥3 at 6, 12, and 24 months had no differences to those without pain in baseline TSP (6 months: d = −0.01, *P* = 0.977; 12 months: d = −0.44, *P* = 0.133; 24 months: d = −0.38, *P* = 0.195) or baseline CPM PTT (6 months: d = −0.32, *P* = 0.263; 12 months: d = −0.33, *P* = 0.218; 24 months: d = 0.16, *P* = 0.553). Individuals with pain ≥3 at 6 and 12 months had no differences to those without pain in baseline CPM PDT effect (6 months: d = −0.10, *P* = 0.736, 12 months: d = 0.07, *P* = 0.807), but those with persistent pain at 24 months had impaired CPM according to the CPM PDT effect (d = 0.67, *P* = 0.015) compared with those without pain.

In mixed-effects regression models, associations between baseline QST measures and subsequent pain were modest and inconsistent. Lower baseline trapezius PPT and a smaller CPM PDT effect both approached significance (trapezius PPT average marginal effects [AME] = −0.51, 95% CI: −1.02 to 0.01, *P* = 0.054; CPM PDT effect AME = −0.49, 95% CI: −0.97 to 0.00, *P* = 0.051), whereas TSP and CPM PTT effect were not associated (TSP [AME = 0.09, 95% CI: −0.47 to 0.65, *P* = 0.756]), CPM PTT effect (AME = −0.01, 95% CI: −0.52 to 0.50, *P* = 0.964).

## 4. Discussion

In this longitudinal study of patients with IA followed from diagnosis, using a comprehensive patient assessment incorporating joint ultrasound, questionnaires, and QST, we characterised the inflammatory, sensory, and psychological features associated with the pain reported by our patients. Half of the patients continued to report clinically relevant pain (NRS score equal to or greater than 3) at 24 months despite a significant reduction in joint inflammation. This builds on our observation that features of centrally mediated pain are present at diagnosis,^35^ and extends the limited longitudinal sensory data available for IA cohorts assessed from diagnosis.

The early pain reduction observed across the cohort likely reflects the direct effects of anti-inflammatory treatments in controlling peripheral inflammation, with secondary benefits including reductions in psychological distress and improvements in sleep, as captured by the PROMs. These findings are consistent with previous large national surveys reporting reductions in psychological distress after IA diagnosis and treatment initiation.^47^ Specifically, in our study, anti-inflammatory treatment reduced affected joint inflammation comparably across patients. Interestingly, these improvements were observed in patients who had reduced pain ratings and in those who developed persistent pain. This highlights a key message of our findings: in our cohort, pain persistence was not necessarily explained by inadequate control of inflammation. Instead, persistent pain ratings of ≥3 were associated with higher baseline and longitudinal scores for features of centrally mediated pain (according to fibromyalgia severity and painDETECT scores), somatic symptom burden (PHQ-15), and psychological distress (PHQ-9 and GAD-7). The convergence across these conceptually related but distinct measures of features associated with centrally mediated pain gives further strength to our findings. Our mixed-effects linear regression models identified these baseline PROMs of centrally mediated pain, but not inflammation, as predictors of subsequent persistent pain. Together, these findings highlight that even when inflammation is reduced in the early stages of disease,^35^ features associated with centrally mediated pain are strongly associated with persistent pain in inflammatory arthritis.

Although the contribution of both inflammatory and noninflammatory mechanisms to pain in established IA is well recognised and reviewed elsewhere,^2,20,31,33,44^ direct comparison with existing literature is limited by the relatively small number of longitudinal studies from diagnosis in IA.^6,12,15–17^ Available studies report similar features associated with persistent pain.^16,17^ Using data from 3 large RA cohorts, including one in early IA (ERAN, BSRBR, and BSRBR-NB), McWilliams and colleagues identified distinct pain trajectories based on SF-36 bodily pain scores. In all 3 cohorts, poorer functional status as measured by the Health Assessment Questionnaire (HAQ) and a history of smoking were associated with a higher likelihood of following the persistent pain trajectory.^17^ A separate study used machine learning analysis of 789 patients followed for 5 years from diagnosis; it identified patient global assessment (PGA) and HAQ scores at 3 months as the strongest predictors of later persistent pain.^16^ Building on this, Lee and colleagues, in a prospective multicentre cohort of patients with newly diagnosed active RA, demonstrated that nonarticular pain, both regional and widespread, was independently associated with worse pain interference and multiple other PROMIS-29 domains over the first year of disease.^22^ Other studies have longitudinally assessed patients with established IA, rather than early disease. Those using the same PROMs align with our findings that higher painDETECT scores^40^ and fibromyalgia symptom severity scores^10^ as well as anxiety and depression^5^ are associated with worse disease activity and quality of life.

In our study, QST was used to probe spinal transmission and modulatory processes. Group-level values for sensory markers of centrally mediated pain—PPT at a remote site (trapezius), TSP, and CPM outcomes—remained unchanged over the 24-month study period, despite patients receiving anti-inflammatory therapy and showing reduced peripheral inflammation at affected joints. Although average baseline CPM effects were lower than those in healthy individuals,^35^ associations between baseline QST markers and persistent pain were modest and inconsistent. Lower baseline PPT, particularly at the trapezius, was modestly associated with persistent pain at several time points and approached significance in our regression models. This finding is consistent with prior cross-sectional reports linking pain sensitivity quantified by reduced nonarticular PPT with worse disease outcomes and pain severity in IA.^38^ However, the dynamic QST paradigms (TSP and CPM) showed no association with persistent pain in this cohort. The significance of TSP and CPM outcomes in IA, where well-established thresholds for clinically meaningful change are lacking,^13,25^ is yet to be determined. A recent study by our group found that cuff-algometry CPM effects in healthy individuals have poor test–retest reliability (ICC = 0.25-0.37), with 38% of participants switching responder classification between sessions,^38^ and a recent meta-analysis reported poor between-session reliability for TSP.^3^ Furthermore, there is large interindividual variability in these measures even among healthy controls with only one-third (approximately) of pain-free individuals demonstrating “antinociceptive” (facilitatory TSP and nonresponder/facilitator CPM) profiles.^27^ Systematic reviews exploring QST measures as predictors of persistent pain have been limited by heterogeneous methodologies and inconsistent results.^21,41^ Although QST measures, specifically TSP and CPM, have been longitudinally shown to predict MSK^9^ and chronic postoperative^26^ pain, the effect sizes are moderate and there are few such studies in IA.^4,11^ Summarising, the utility of TSP and CPM as one-off prognostic biomarkers in IA is yet to be established.

Our cohort was representative of routine IA clinic populations, with 56% female participants and ethnically inclusive representation (55% identified as White British). Patients were recruited early in their disease process, with a mean disease duration of 1.2 months.^35^ Follow-up rates were acceptable, with 92% of participants completing at least one follow-up assessment. Participants underwent repeated assessments at multiple time points over a 2-year period. We used mixed-effects modelling to account for within-subject variability and incomplete data across time points, and the composite scores for inflammation and centrally mediated pain avoided collinearity in subsequent modelling.

On limitations, our sample size limited statistical power for certain analyses, particularly multivariable logistic regression analyses, and raises the possibility of overfitting in models incorporating multiple predictors. To mitigate this, we first used simple between-group comparisons (*t* tests with effect size estimation) to identify variables differing between those with and without persistent pain at each time point, and only then carried forward variables into mixed-effects models. We also performed linear regression with repeated measures to increase power and performed careful stepwise selection of variables. Strong and consistent associations for individual PROM variables provide reassurance against overfitting. Findings should be interpreted in the context of the cohort size, and confirmation in larger samples will be important. The study may have had limited power to detect modest QST–pain associations, and the borderline findings for trapezius PPT and the CPM PDT effect (both *P* ≈ 0.05) would benefit from confirmation in larger cohorts. Attrition occurred, leaving the possibility of responder or attrition bias. Participants lost to follow-up had higher baseline DAS28 tender joint counts and PHQ-9 scores (features associated with persistent pain) than those who completed 24-month follow up, so any bias from missing data in the predictive models is likely to underestimate the true associations. The inclusion criteria of a pain score ≥3 was selected based on prior literature^8^ and because some of our measures (eg, painDETECT) required the presence of pain for assessment. To maintain consistency, and in line with previous studies that have used cutoffs between 2 and 4,^8,18,37^ we also applied this threshold when defining “clinically relevant persistent pain.” This approach limits the generalisability of our findings to patients with lower pain levels. Finally, although DMARD treatment exposure was similar in the persistent and resolved pain groups, in larger or more heterogeneous cohorts' variation in treatment intensity, response or escalation could potentially influence the predictive value of PROMs.

Summarising, the fact that half of our IA patients continued to experience clinically meaningful pain 24 months after diagnosis, with 25% experiencing no pain improvement despite anti-inflammatory therapies reducing inflammation at the affected joint, indicates an unmet need in pain management. Our results indicate that features associated with centrally mediated pain are strongly linked to pain persistence, reinforcing the importance of moving beyond an inflammation-targeting-only model of pain management in IA and supporting previous work emphasising the role of central modulation in IA.^42^ Our findings have several practical implications for clinical care. First, they support routinely screening for features associated with centrally mediated pain at IA diagnosis. Second, they caution against the assumption that persistent pain reflects inadequate control of inflammation, and against escalating immunosuppression in response to pain that is not inflammatory in origin. Third, the observation that fibromyalgia-like features were not fixed and improved over time suggests that these features may be modifiable with targeted intervention, alongside disease-modifying treatment. Fourth, identifying centrally mediated features early offers an opportunity to reframe conversations with patients about why pain may persist despite effective control of inflammation, setting realistic expectations about treatment trajectory. Future research must focus on validating stratification tools that include centrally mediated pain markers, identifying effective targeted interventions, and testing these in stratified trials. Longer-term follow-up will also be important to understand the trajectory of peripheral and central nervous system pain features over time.

## Conflict of interest statement

The authors have no conflict of interest to declare.

## Supplemental digital content

Supplemental digital content associated with this article can be found online at https://links.lww.com/PAIN/C559.

## Supplementary Material
