# Supplementary material for: Centrally mediated pain features predict persistent pain in early inflammatory arthritis: a 2-year prospective study
Source: Pain. 2026 Sep 2;167(10):2365–74. doi: 10.1097/j.pain.0000000000004099 (PMC13580213; doi:10.1097/j.pain.0000000000004099)
Supplement: Supplementary file 1 [file jop-167-2365-s001.pdf]

Supplementary material

Supplementary Data S1: Recruitment criteria and Dynamic QST protocols

Participants were recruited from the rheumatology outpatient department at Guy's Hospital, London, between February and November 2022. Eligible individuals were those who reported a total numerical rating scale (NRS) pain score of at least 3 (on a 0–10 scale), had a clinician-confirmed diagnosis of peripheral inflammatory joint disease, and had experienced symptom onset within the preceding 12 months. Recruitment occurred as early as possible after diagnosis. Exclusion criteria included being under 18 years of age, inability or unwillingness to provide written informed consent, inability to comply with study procedures, pregnancy or breastfeeding, use of immunosuppressive treatments not related to inflammatory arthritis, current or recent (within 90 days) exposure to investigational medications, and a history of severe peripheral vascular disease or peripheral neuropathy. Participants were also asked to withhold opioids, gabapentin, and pregabalin for 24 hours prior to assessment.

Temporal summation of pain (TSP) using cuff pressure algometer

The test leg cuff was inflated for 1 second, repeated 10 times at 1-second intervals, at a pressure equivalent to 100% of the participant's pressure pain tolerance in that leg. Participants were asked to rate the pain experienced during each compression using a visual analog scale (VAS). TSP was determined by calculating the ratio of VAS III (mean VAS for stimulations 8-10) to VAS I (mean VAS for stimulations 1-4), where TSP is considered to be facilitated if the ratio exceeds 2.48[1].

Conditioned pain modulation (CPM) using cuff pressure algometer

Two blood pressure cuff-like devices placed around the widest part of the gastrocnemius muscle on the lower leg act as the test stimulus (dominant leg) and conditioning stimulus (non-dominant leg). These were inflated at a computer-controlled steady rate of 1kPa/second using compressed air. The subject was given a visual analogue scale bar and asked to begin to slide the bar at first sensation of pain. The pressure value when the patient rates 1cm on the VAS is the pain detection threshold (PDT) and when the patient can no longer tolerate the pressure they press the stop button to immediately deflate the cuff (pain tolerance threshold, PTT). Firstly, the PDT and PTT were assessed on the test leg, and then CPM was assessed as the PDT and PTT in the test leg with the conditioning leg concurrently inflated (at 70% of the PTT). CPM response was defined as PPT CPM effect = $PPT_{(CPM)}$- $PPT_{(Dom)}$ and PTT CPM effect = $PTT_{(CPM)}$- $PTT_{(Dom)}$. Participants were stratified as responders (CPM effect > 20% of the baseline cPPT) or non-responders (CPM effect ≤20% of baseline cPPT)[2]

Characteristic	Value (n=66)
**Demographics**	
Age, years, mean (SD; range)	50 (15; 19–86)
Female, n (%)	37 (56)
Ethnicity, n (%)	
White British	36 (55)
Black or Black British	11 (17)
Asian or Asian British	9 (14)
Other	10 (15)
Smoker (current or previous), n (%)	26 (39)
Alcohol use (current or previous), n (%)	40 (61)
Unemployed, n (%)	11 (17)
BMI, kg/m², mean (SD)	27.0 (5.6)
**Disease characteristics**	
Diagnosis, n (%)	
Rheumatoid arthritis	54 (82)
Psoriatic arthritis	10 (15)
Spondyloarthritis	2 (3)
Seropositive (RhF or anti-CCP), n (%)	42 (64)
Time since diagnosis, months, mean (SD)	1.2 (2.2)
Symptom duration, months, mean (SD)	6.3 (3.1)
DAS28-CRP, mean (SD)	3.9 (1.0)
CRP, mg/L, mean (SD)	9.5 (13)
28-TJC, mean (SD)	6.4 (5.1)
28-SJC, mean (SD)	2.4 (2.6)
DAS28-CRP category, n (%)	
Remission	10 (15)
Low disease activity	10 (15)
Moderate disease activity	40 (61)
High disease activity	6 (9)
**Musculoskeletal ultrasound, n (%)**	
≥1 GS-positive joint	56 (85)
≥1 PD-positive joint	37 (56)
≥1 GS-positive, ≥1 PD-positive, or ≥1 SJC	65 (99)
≥1 PD-positive or ≥1 SJC	54 (82)
**Medications, n (%)**	
Paracetamol or NSAID	19 (29)
Codeine-based analgesic	6 (9)

Characteristic	Value (n=66)
Amitriptyline	4 (6)
csDMARD	38 (58)
Corticosteroid (IM or PO)	40 (61)
**Quality of life**	
MSK-HQ, mean (SD)	29 (11)
**Mental health**	
GAD-7, mean (SD)	7.4 (6.0)
Any anxiety (≥5), n (%)	41 (62)
Mild (5–9), n (%)	22 (33)
Moderate (10–14), n (%)	8 (12)
Severe (≥15), n (%)	11 (17)
PHQ-9, mean (SD)	8.3 (6.3)
Any depression (≥5), n (%)	44 (67)
Mild (5–9), n (%)	18 (27)
Moderate (10–14), n (%)	15 (23)
Moderately severe (15–19), n (%)	8 (12)
Severe (≥20), n (%)	3 (5)
PHQ-15, mean (SD)	8.9 (5.3)
Any somatic symptoms (≥5), n (%)	53 (80)
Mild (5–9), n (%)	29 (44)
Moderate (10–14), n (%)	15 (23)
Severe (≥15), n (%)	9 (14)
**Pain**	
Overall body pain (NRS 0–10), mean (SD)	5.4 (2.1)
Affected joint pain (NRS 0–10), mean (SD)	5.1 (2.5)
**Widespread pain and fibromyalgia features**	
Widespread Pain Index (WPI), mean (SD)	5.6 (2.8)
WPI ≥7, n (%)	21 (32)
Fibromyalgia (2016 ACR criteria), n (%)	15 (23)
Fibromyalgia severity (WPI + SSS), mean (SD)	10.9 (4.9)
Tender–swollen joint count difference ≥7, n (%)	15 (23)
**Neuropathic-like pain**	
painDETECT, mean (SD)	12.7 (7.7)
Likely neuropathic-like (≥19), n (%)	15 (23)
Possible neuropathic-like (13–18), n (%)	12 (18)
Unlikely neuropathic-like (≤12), n (%)	39 (59)
**Quantitative sensory testing, mean (SD)**	

Characteristic	Value (n=66)
PPT, affected joint (kg/cm²)	2.22 (1.5)
PPT, right wrist (kg/cm²)	2.5 (1.2)
PPT, left wrist (kg/cm²)	2.7 (1.7)
PPT, right trapezius (kg/cm²)	3.3 (1.9)
PPT, left trapezius (kg/cm²)	3.1 (1.7)
TSP ratio	1.9 (1.2)
CPM PDT effect (kPa)	2.6 (8.3)
CPM PTT effect (kPa)	3.4 (6.7)

Supplementary Table S1: Full table showing patient demographics, diagnosis (at 6 months), patient reported outcomes measures and quantitative sensory testing. (n=66). Abbreviations: BMI (Body mass index), EIA (Early inflammatory arthritis), RA (Rheumatoid arthritis), PsA (Psoriatic arthritis), SpA (Spondylarthritis), RhF (Rheumatoid factor), CCP (cyclic citrullinated peptide), IM (Intra-muscular), PO (Oral), IV (Intra-venous), DMARDS (Disease modifying anti-rheumatic drugs), MSK HQ (Musculoskeletal health questionnaire), GAD 7 (Generalised anxiety disorder 7 questionnaire), PHQ9 (Patient health questionnaire 9), PHQ15 (Patient health questionnaire 15).

Variable	Baseline mean (SD)	6 months mean (SD)	12 months mean (SD)	24 months mean (SD)
**Disease activity and quality of life**				
DAS28-CRP	3.9 (1.0)	–	2.9 (1.1)	–
MSK-HQ	29.0 (11.2)	35.9 (13.1)	35.9 (13.1)	39.6 (12.1)
Fatigue (VAS)	46.7 (27.6)	–	42.4 (31.1)	–
**Inflammatory markers**				
CRP, mg/L	9.5 (13.3)	–	4.0 (5.3)	–
28-SJC	2.4 (2.6)	–	0.7 (1.3)	–
HRUS GS/PD total	9.1 (10.2)	–	7.5 (9.5)	–
**Features associated with centrally mediated pain**				
Fibromyalgia severity	10.9 (4.9)	9.0 (5.3)	8.4 (5.2)	8.0 (5.0)
painDETECT	12.7 (7.7)	9.8 (7.1)	10.6 (7.1)	9.8 (7.8)
Tender–swollen joint count difference	4.0 (5.2)	–	2.5 (2.8)	–
**Mental health**				
PHQ-9	8.3 (6.3)	6.3 (6.2)	6.0 (6.6)	5.9 (6.8)
GAD-7	7.4 (6.0)	5.0 (5.5)	5.9 (6.0)	4.6 (5.3)

Variable	Baseline mean (SD)	6 months mean (SD)	12 months mean (SD)	24 months mean (SD)
PHQ-15	8.9 (5.3)	6.7 (4.7)	7.2 (4.5)	6.6 (5.0)
**Quantitative sensory testing**				
PPT, target joint (kg/cm^2)	2.2 (1.5)	–	2.9 (1.7)	–
PPT, right wrist (kg/cm^2)	2.5 (1.2)	–	3.5 (1.7)	–
PPT, left wrist (kg/cm^2)	2.7 (1.7)	–	3.4 (1.7)	–
PPT, right trapezius (kg/cm^2)	3.3 (1.9)	–	3.4 (2.2)	–
PPT, left trapezius (kg/cm^2)	3.1 (1.7)	–	3.4 (1.9)	–
TSP ratio	1.9 (1.2)	–	2.9 (6.6)	–
CPM PDT effect (kPa)	2.6 (8.3)	–	4.9 (8.4)	–
CPM PTT effect (kPa)	3.4 (6.7)	–	2.6 (8.7)	–

Supplementary Table S2. Observed (Raw) Means and Standard Deviations for Key Outcomes Over Time Unadjusted means (standard deviations) shown for each timepoint. MSK-HQ: Musculoskeletal Health Questionnaire; DAS28: Disease Activity Score (28 joints); SJC: Swollen Joint Count; CRP: C-reactive protein; EGA: Evaluator Global Assessment; HRUS: High-Resolution Ultrasound; GS: Grey Scale; PD: Power Doppler; T-SJC: Tender minus Swollen Joint Count; FM: Fibromyalgia; PHQ-9: Patient Health Questionnaire–Depression; PHQ-15: Patient Health Questionnaire–Somatic Symptoms; GAD-7: Generalised Anxiety Disorder Scale

Variable	Baseline mean (SE)	6 months mean (SE)	12 months mean (SE)	24 months mean (SE)	Significant pairwise comparisons
**Disease activity and quality of life**					
MSK-HQ	29.0 (1.5)	35.1 (1.6)	35.6 (1.57)	39.3 (1.61)	0 vs 6, 12, 24; 6 vs 24
Fatigue (VAS)	46.7 (3.6)	–	42.3 (3.9)	–	NS
**Inflammatory markers**					
CRP, mg/L	9.5 (1.3)	–	4.6 (1.4)	–	0 vs 12
28-SJC	2.4 (0.26)	–	0.7 (0.27)	–	0 vs 12
EGA	43.2 (2.7)	–	30.6 (3)	–	0 vs 12

Variable	Baseline mean (SE)	6 months mean (SE)	12 months mean (SE)	24 months mean (SE)	Significant pairwise comparisons
HRUS GS/PD total	9.1 (1.2)	–	7.3 (1.3)	–	NS
**Features associated with centrally mediated pain**					
Fibromyalgia severity	10.9 (0.6)	9.4 (0.7)	8.7 (0.6)	8.2 (0.7)	0 vs 6, 12, 24
painDETECT	12.7 (0.9)	10.5 (1.0)	10.8 (1.0)	10 (1.0)	0 vs 24
**Mental health**					
PHQ-9	8.3 (0.8)	7.0 (0.9)	6.5 (0.8)	6.6 (0.9)	0 vs 12; 0 vs 24
GAD-7	7.4 (0.7)	5.8 (0.8)	6.1 (0.8)	5.1 (0.8)	0 vs 6; 0 vs 24
PHQ-15	8.9 (0.6)	7.1 (0.7)	7.6 (0.6)	6.9 (0.7)	0 vs 6, 12, 24
**Quantitative sensory testing**					
PPT, target joint	2.2 (0.2)	–	2.9 (0.2)	–	0 vs 12
PPT, right wrist	2.6 (0.2)	–	3.5 (0.2)	–	0 vs 12
PPT, left wrist	2.7 (0.2)	–	3.3 (0.2)	–	0 vs 12
PPT, right trapezius	3.3 (0.2)	–	3.3 (0.3)	–	NS
PPT, left trapezius	3.3 (0.2)	–	3.3 (0.2)	–	NS
TSP ratio	1.9 (0.6)	–	2.9 (0.7)	–	NS
CPM PDT effect	2.6 (1.0)	–	4.7 (1.1)	–	NS
CPM PTT effect	3.4 (0.9)	–	2.5 (1.1)	–	NS

Supplementary Table S3. Modelled Estimated Means (SE) for Key Outcomes Over Time from Mixed-Effects Regression Models Estimated marginal means and standard errors (SEs) derived from mixed-effects linear regression models, adjusted for repeated measures by participant NS= non-significant. MSK-HQ: Musculoskeletal Health Questionnaire; DAS28: Disease Activity Score (28 joints); SJC: Swollen Joint Count; CRP: C-reactive protein; EGA: Evaluator Global Assessment; HRUS: High-Resolution Ultrasound; GS: Grey Scale; PD: Power Doppler; FM: Fibromyalgia; PHQ-9: Patient Health Questionnaire–Depression; PHQ-15: Patient Health Questionnaire–Somatic Symptoms; GAD-7: Generalised Anxiety Disorder

Variable	6 months			12 months			24 months		
	Mean diff. (95% CI)	p	Cohen's d (95% CI)	Mean diff. (95% CI)	p	Cohen's d (95% CI)	Mean diff. (95% CI)	p	Cohen's d (95% CI)
**Demographics and disease activity**									
Age	2.71 (−5.50 to 10.93)	0.51	0.19 (−0.37 to 0.74)	9.37 (1.77 to 16.97)	0.017	0.66 (0.12 to 1.19)	0.11 (−7.84 to 8.06)	0.978	0.01 (−0.51 to 0.53)
BMI	−2.78 (−6.11 to 0.55)	0.10	−0.47 (−1.03 to 0.09)	−2.21 (−5.19 to 0.77)	0.143	−0.39 (−0.92 to 0.13)	−2.88 (−5.86 to 0.10)	0.058	−0.51 (−1.04 to 0.02)
Symptom duration	−1.11 (−2.89 to 0.68)	0.218	−0.35 (−0.91 to 0.21)	−1.31 (−2.92 to 0.30)	0.110	−0.43 (−0.95 to 0.10)	−0.15 (−1.84 to 1.53)	0.854	−0.05 (−0.57 to 0.47)
Disease duration	0.75 (−0.61 to 2.12)	0.275	0.31 (−0.25 to 0.87)	−0.05 (−1.08 to 0.98)	0.922	−0.03 (−0.55 to 0.49)	1.12 (−0.10 to 2.33)	0.071	0.49 (−0.04 to 1.01)
DAS28-CRP	−0.45 (−1.02 to 0.12)	0.118	−0.45 (−1.01 to 0.11)	−0.32 (−0.85 to 0.21)	0.234	−0.32 (−0.84 to 0.21)	−0.39 (−0.91 to 0.13)	0.136	−0.40 (−0.92 to 0.13)
Fatigue	−26.95 (−41.18 to −12.73)	0.001	−1.08 (−1.67 to −0.48)	−21.76 (−35.41 to −8.10)	0.002	−0.85 (−1.39 to −0.30)	−19.16 (−32.96 to −5.37)	0.007	−0.74 (−1.27 to −0.20)
Pain	−0.78 (−2.01 to 0.46)	0.212	−0.36 (−0.92 to 0.20)	−0.75 (−1.86 to 0.37)	0.186	−0.35 (−0.88 to 0.17)	−1.42 (−2.50 to −0.33)	0.012	−0.69 (−1.22 to −0.15)
Joint pain	−0.70 (−2.08 to 0.67)	0.310	−0.29 (−0.85 to 0.27)	−0.69 (−1.98 to 0.60)	0.292	−0.28 (−0.80 to 0.24)	−1.10 (−2.38 to 0.19)	0.093	−0.45 (−0.98 to 0.08)
PGA	−16.50 (−29.12 to −3.89)	0.011	−0.74 (−1.31 to −0.17)	−15.25 (−27.19 to −3.30)	0.013	−0.68 (−1.21 to −0.14)	−19.66 (−31.21 to −8.10)	0.001	−0.90 (−1.45 to −0.35)
28-TJC	−0.65 (−3.34 to 2.04)	0.629	−0.14 (−0.69 to 0.42)	0.21 (−2.26 to 2.69)	0.863	0.05 (−0.47 to 0.56)	−1.96 (−4.39 to 0.46)	0.111	−0.43 (−0.95 to 0.10)
MSK-HQ	8.72 (2.80 to 14.64)	0.005	0.84 (0.26 to 1.41)	2.98 (−2.96 to 8.91)	0.319	0.27 (−0.26 to 0.79)	8.30 (2.74 to 13.87)	0.004	0.79 (0.25 to 1.33)
**Features associated with centrally mediated pain**									
Fibromyalgia severity	−4.32 (−6.72 to −1.92)	0.001	−1.02 (−1.61 to −0.43)	−2.26 (−4.73 to 0.22)	0.073	−0.48 (−1.01 to 0.04)	−3.35 (−5.76 to −0.95)	0.007	−0.74 (−1.27 to −0.20)
painDETECT	−6.75 (−10.45 to −3.06)	0.001	−1.04 (−1.63 to −0.45)	−6.63 (−10.28 to −2.98)	<0.001	−0.96 (−1.51 to −0.41)	−8.03 (−11.36 to −4.69)	<0.001	−1.28 (−1.84 to −0.70)

Variable	6 months			12 months			24 months		
	**Mean diff. (95% CI)**	**p**	**Cohen's d (95% CI)**	**Mean diff. (95% CI)**	**p**	**Cohen's d (95% CI)**	**Mean diff. (95% CI)**	**p**	**Cohen's d (95% CI)**
Tender–swollen joint count diff.	−0.50 (−3.20 to 2.22)	0.700	0.10 (−0.46 to 0.66)	1.17 (−2.63 to 2.06)	0.800	0.06 (−0.46 to 0.58)	−2.12 (−4.40 to 0.17)	0.070	0.49 (−0.04 to 1.02)
**Mental health**									
GAD-7	−4.27 (−7.18 to −1.37)	0.005	−0.84 (−1.41 to −0.25)	−4.51 (−7.39 to −1.63)	0.003	−0.83 (−1.37 to −0.29)	−4.46 (−7.32 to −1.61)	0.003	−0.83 (−1.37 to −0.28)
PHQ-15	−4.76 (−7.19 to −2.33)	0.001	−1.11 (−1.71 to −0.51)	−4.66 (−6.96 to −2.37)	<0.001	−1.08 (−1.63 to −0.52)	−3.70 (−6.09 to −1.31)	0.003	−0.82 (−1.36 to −0.28)
PHQ-9	−5.35 (−8.46 to −2.23)	0.001	−0.98 (−1.56 to −0.39)	−3.85 (−6.89 to −0.81)	0.014	−0.67 (−1.21 to −0.14)	−3.08 (−6.15 to 0.00)	0.050	−0.53 (−1.06 to 0.00)
**Inflammatory markers**									
CRP	5.26 (−1.79 to 12.30)	0.140	0.42 (−0.14 to 0.98)	1.76 (−3.37 to 6.88)	0.495	0.18 (−0.34 to 0.70)	5.13 (−1.26 to 11.52)	0.114	0.43 (−0.10 to 0.95)
28-SJC	−0.15 (−1.54 to 1.24)	0.826	−0.06 (−0.62 to 0.49)	0.50 (−0.84 to 1.83)	0.458	0.20 (−0.32 to 0.72)	0.16 (−1.20 to 1.52)	0.816	0.06 (−0.46 to 0.58)
EGA	−10.58 (−22.18 to 1.03)	0.073	−0.52 (−1.08 to 0.05)	−5.87 (−16.52 to 4.78)	0.275	−0.29 (−0.81 to 0.23)	−4.37 (−15.03 to 6.28)	0.414	−0.22 (−0.74 to 0.30)
HRUS Total GS/PD	−1.74 (−7.30 to 3.82)	0.533	−0.18 (−0.73 to 0.38)	2.37 (−3.11 to 7.85)	0.391	0.23 (−0.29 to 0.75)	2.47 (−3.04 to 7.98)	0.372	0.24 (−0.28 to 0.76)
**Quantitative sensory testing**									
PPT, target joint	0.34 (−0.61 to 1.29)	0.481	0.20 (−0.36 to 0.76)	0.87 (0.04 to 1.69)	0.040	0.56 (0.03 to 1.08)	0.84 (0.01 to 1.67)	0.047	0.54 (0.01 to 1.06)
PPT, right wrist	0.73 (0.05 to 1.41)	0.036	0.65 (0.04 to 1.25)	0.19 (−0.48 to 0.86)	0.581	0.16 (−0.40 to 0.71)	0.39 (−0.28 to 1.07)	0.245	0.33 (−0.23 to 0.88)
PPT, left wrist	0.83 (−0.28 to 1.94)	0.141	0.45 (−0.15 to 1.05)	0.21 (−0.83 to 1.25)	0.682	0.12 (−0.44 to 0.68)	1.02 (0.03 to 2.01)	0.043	0.59 (0.02 to 1.15)
PPT, right trapezius	0.46 (−0.67 to 1.59)	0.421	0.23 (−0.33 to 0.79)	0.70 (−0.32 to 1.73)	0.174	0.36 (−0.16 to 0.89)	1.11 (0.11 to 2.10)	0.030	0.59 (0.06 to 1.12)

Variable	6 months			12 months			24 months		
	Mean diff. (95% CI)	p	Cohen's d (95% CI)	Mean diff. (95% CI)	p	Cohen's d (95% CI)	Mean diff. (95% CI)	p	Cohen's d (95% CI)
TSP ratio	−0.01 (−0.84 to 0.82)	0.977	−0.01 (−0.61 to 0.59)	−0.39 (−0.89 to 0.12)	0.133	−0.44 (−1.02 to 0.14)	−0.49 (−1.23 to 0.26)	0.195	−0.38 (−0.95 to 0.19)
CPM PDT effect	−0.79 (−5.43 to 3.86)	0.736	−0.10 (−0.65 to 0.46)	0.52 (−3.69 to 4.72)	0.807	0.07 (−0.46 to 0.58)	5.16 (1.05 to 9.26)	0.015	0.67 (0.13 to 1.20)
CPM PTT effect	−1.99 (−5.51 to 1.53)	0.263	−0.32 (−0.88 to 0.24)	−1.98 (−5.16 to 1.20)	0.218	−0.33 (−0.85 to 0.20)	0.96 (−2.27 to 4.20)	0.553	0.16 (−0.36 to 0.68)

Supplementary Table S4: Baseline predictors of pain at 6, 12, and 24 months. Mean differences (95% CI), p-values, and Cohen's *d* values are shown for individuals with and without persistent pain (NRS ≥3) at each timepoint. Fatigue, psychological distress (GAD-7, PHQ-9, PHQ-15), and PROMs of features associated with centrally mediated pain (painDETECT, fibromyalgia severity) showed the largest and most consistent differences. Inflammatory markers (CRP, DAS28, HRUS) and QST measures showed minimal or inconsistent differences across timepoints. Cells with $p < 0.05$ are shaded blue. Cohen's d effect-size cells are shaded by magnitude using standard thresholds: light green for small (|d| 0.2–<0.5), medium green for medium (|d| 0.5–<0.8), and dark green for large (|d| ≥ 0.8) effects.

Medication category	Baseline (n=66)	6 months (n=53)	12 months (n=58)	24 months (n=57)
Paracetamol or NSAID	19 (29)	11 (21)	8 (14)	7 (12)
Codeine-based analgesic	6 (9)	4 (8)	4 (7)	3 (5)
Amitriptyline	4 (6)	2 (4)	2 (3)	2 (9%)
csDMARD	38 (58)	48 (91)	54 (93)	54 (95)
Corticosteroid (IM or PO)	40 (61)	3 (6)	7 (12)	5 (9)
bDMARD/tsDMARD	0 (0)	1 (2)	13 (22)	13 (23)

Supplementary Table S5. Ongoing medications by study visit. Values are n (%) of participants attending each visit who were receiving each medication class. csDMARD = conventional synthetic disease-modifying antirheumatic drug; bDMARD = biologic DMARD; tsDMARD = targeted synthetic DMARD; IM = intramuscular; PO = per oral; NSAID = non-steroidal anti-inflammatory drug.

	Persistent pain (≥3 at 24 months)				Resolved pain (<3 at 24 months)			
Medication category	Baseline (n=28)	6 m (n=21)	12 m (n=28)	24 m (n=28)	Baseline (n=29)	6 m (n=29)	12 m (n=27)	24 m (n=29)
Paracetamol or NSAID	9 (32)	7 (33)	6 (21)	5 (18)	8 (28)	4 (14)	2 (7)	2 (7)
Codeine-based analgesic	4 (14)	3 (14)	3 (11)	2 (7)	1 (3)	1 (3)	1 (4)	1 (3)
Amitriptyline	3 (11)	2 (10)	2 (7)	2 (7)	0 (0)	0 (0)	0 (0)	0 (0)
csDMARD	17 (61)	19 (90)	25 (89)	26 (93)	17 (59)	26 (90)	26 (96)	28 (97)
Corticosteroid (IM or PO)	15 (54)	0 (0)	4 (14)	3 (11)	20 (69)	3 (10)	3 (11)	2 (7)
bDMARD/tsDMARD	0 (0)	0 (0)	7 (25)	7 (25)	0 (0)	1 (3)	5 (19)	6 (21)

Supplementary Table S6. Ongoing medications by study visit, stratified by pain status at 24 months. Values are n (%) of participants attending each visit who were receiving each medication class, stratified by pain status at 24 months (NRS ≥3 = persistent pain; NRS <3 = resolved pain). Baseline n (%) values for persistent and resolved groups do not sum exactly to the overall baseline values in Supplementary Table S5 because 9 participants enrolled at baseline did not have pain data at 24 months and could not be classified. Medication classes are defined as in Supplementary Table S5.

Baseline characteristic	Lost to follow-up (n = 9)	Completed 24m (n = 57)	*p*
Demographics			
Age, years	51.6 (19.0)	49.8 (14.8)	0.758
BMI, kg/m²	26.1 (5.2)	26.8 (5.8)	0.737
Symptom duration, months	5.3 (2.5)	6.4 (3.1)	0.328
Disease activity & inflammation			
DAS28	4.2 (1.1)	3.8 (1.0)	0.321
Tender joint count (DAS28)	9.7 (7.1)	5.9 (4.6)	**0.040**

Baseline characteristic	Lost to follow-up (n = 9)	Completed 24m (n = 57)	*p*
Swollen joint count (DAS28)	1.9 (3.3)	2.5 (2.5)	0.541
CRP, mg/L	12.7 (19.6)	9.0 (12.2)	0.443
US power Doppler (total)	2.0 (3.7)	3.5 (4.8)	0.393
Inflammation composite (factor)	-0.3 (1.0)	0.0 (0.9)	0.345
Pain & function			
Overall pain (NRS 0–10)	6.0 (1.7)	5.3 (2.1)	0.393
Joint pain (NRS 0–10)	4.2 (2.3)	5.2 (2.5)	0.256
MSK-HQ	26.6 (11.6)	29.4 (11.2)	0.483
Fatigue (VAS 0–100)	51.1 (29.3)	46.0 (27.5)	0.607
Centrally mediated pain features			
painDETECT	13.3 (9.7)	12.6 (7.4)	0.801
Fibromyalgia severity	13.4 (4.9)	10.5 (4.8)	0.097
Widespread Pain Index	7.2 (2.5)	5.3 (2.8)	0.064
Symptom Severity Scale	6.2 (3.3)	5.2 (3.0)	0.353
Central composite (factor)	0.4 (1.1)	-0.1 (0.8)	0.161
Psychological distress			
PHQ-9	12.3 (7.6)	7.6 (6.0)	**0.037**
GAD-7	8.8 (7.5)	7.2 (5.8)	0.456
PHQ-15	11.1 (7.9)	8.5 (4.8)	0.177
QST			
Target joint PPT, kg/cm²	2.0 (1.1)	2.3 (1.6)	0.583
Right trapezius PPT	2.7 (1.5) [n=8]	3.4 (1.9)	0.292
Left trapezius PPT	2.4 (1.3)	3.3 (1.8)	0.185
Right wrist PPT	2.0 (1.1)	2.6 (1.2)	0.196
Left wrist PPT	2.2 (1.6)	2.8 (1.7)	0.351
TSP	1.8 (0.8)	1.9 (1.3)	0.808
CPM PDT effect	-2.1 (8.3)	3.3 (8.1)	0.069
CPM PTT effect	0.9 (10.2)	3.8 (6.0)	0.230
Demographics & background (categorical)			
Gender, n (%)	Female: 6 (67%)<br>Male: 3 (33%)	Female: 31 (54%)<br>Male: 26 (46%)	0.720
Ethnicity, n (%)	White British: 5 (56%)<br>Other White: 0 (0%)<br>Black/Black British: 2 (22%)	White British: 31 (54%)<br>Other White: 4 (7%)<br>Black/Black British: 9 (16%)	0.475

Baseline characteristic	Lost to follow-up (n = 9)	Completed 24m (n = 57)	*p*
	Asian/Asian British: 2 (22%)<br>Mixed or other: 0 (0%)	Asian/Asian British: 7 (12%)<br>Mixed or other: 6 (11%)	
Diagnosis, n (%)	Rheumatoid arthritis: 4 (44%)<br>Early IA : 5 (56%)<br>Psoriatic arthritis: 0 (0%)<br>Spondyloarthropathy: 0 (0%)	Rheumatoid arthritis: 37 (65%)<br>Early IA : 12 (21%)<br>Psoriatic arthritis: 7 (12%)<br>Spondyloarthropathy: 1 (2%)	0.235
Smoking status, n (%)	Never: 5 (56%)<br>Current: 2 (22%)<br>Previous: 2 (22%)	Never: 35 (61%)<br>Current: 10 (18%)<br>Previous: 12 (21%)	0.887
Alcohol status, n (%)	Current: 5 (56%)<br>Never: 4 (44%)<br>Previous: 0 (0%)	Current: 32 (56%)<br>Never: 22 (39%)<br>Previous: 3 (5%)	1.000
Employment, n (%)	Full-time: 3 (33%)<br>Part-time: 2 (22%)<br>Retired: 1 (11%)<br>Unemployed: 3 (33%)	Full-time: 31 (54%)<br>Part-time: 5 (9%)<br>Retired: 13 (23%)<br>Unemployed: 8 (14%)	0.181
Rheumatoid factor positive, n (%)	Yes: 4 (44%)<br>No: 5 (56%)	Yes: 26 (46%)<br>No: 30 (54%)	1.000
Anti-CCP positive, n (%)	Yes: 7 (78%)<br>No: 2 (22%)	Yes: 21 (38%)<br>No: 35 (63%)	0.065

Supplementary Table S7. Baseline characteristics by 24-month follow-up status Baseline characteristics of participants who completed the 24-month assessment (n = 57) compared with those lost to follow-up (n = 9). Values are mean (SD) for continuous variables and n (%) for categorical variables. p-values from independent t-tests (continuous) and Fisher's exact tests (categorical). Rows highlighted in yellow indicate $p < 0.05$. Abbreviations: BMI, body mass index; CCP, cyclic citrullinated peptide; CPM, conditioned pain modulation; CRP, C-reactive protein; DAS28, Disease Activity Score in 28 joints; GAD-7, Generalised Anxiety Disorder 7-item; IA, inflammatory arthritis; MSK-HQ, Musculoskeletal Health Questionnaire; NRS, numerical rating scale; PDT, pain detection threshold; PHQ-9, Patient Health Questionnaire-9; PHQ-15, Patient Health Questionnaire-15; PPT, pressure pain threshold; PTT, pain tolerance threshold; SD, standard deviation; TSP, temporal summation of pain; US, ultrasound; VAS, visual analogue scale.

1. Graven-Nielsen T, Arendt-Nielsen L. Assessment of mechanisms in localized and widespread musculoskeletal pain. *Nat Rev Rheumatol* 2010; 6: 599–606.

2. Vaegter HB, Graven-Nielsen T. Pain modulatory phenotypes differentiate subgroups with different clinical and experimental pain sensitivity. *Pain* 2016; 157: 1480–1488.
